# Research Status and Prospect of Amphibian Symbiotic Microbiota

**DOI:** 10.3390/ani15070934

**Published:** 2025-03-25

**Authors:** Ziyi Wang, Yuting Wang, Zhirong He, Siyu Wu, Suyue Wang, Na Zhao, Wei Zhu, Jianping Jiang, Supen Wang

**Affiliations:** 1 The Anhui Provincial Key Laboratory of Biodiversity Conservation and Ecological Security in the Yangtze River Basin, College of Life Sciences, Anhui Normal University, Wuhu 241000, China; 2421011735@ahnu.edu.cn (Z.W.); shijianhai333@126.com (Y.W.); 19111701085@ahnu.edu.cn (Z.H.); 19855304469@163.com (S.W.); wangsuyue02@126.com (S.W.); zhaona@ahnu.edu.cn (N.Z.); 2 Chengdu Institute of Biology, Chinese Academy of Sciences, Chengdu 610041, China; zhuwei@cib.ac.cn

**Keywords:** biodiversity, amphibians, symbiotic microbiota, gut microbiota, skin microbiota

## Abstract

Amphibians are the most severely threatened vertebrate group in terms of biodiversity. The microbiota that coexist in a mutualistic relationship with amphibians play a crucial role in shaping their health status, reproductive efficiency, and environmental adaptability. Understanding the relationship between amphibians and microbiota is vital for elucidating the causes of amphibian diseases and developing effective prevention and control techniques, which in turn is significant for enhancing the effectiveness of amphibian diversity conservation. The main findings of this article are as follows: Firstly, it provides an overview of the systematic assessment and analysis methods regarding the importance of amphibians and their symbiotic microbiota, detailing the primary research techniques currently employed. Secondly, it discusses the impacts of environmental and biological factors on the characteristics of amphibian symbiotic microbial communities, including dimensions such as altitude, temperature fluctuations, and host dietary habits. Finally, the future directions of research on amphibian symbiotic microbiota are examined, with five recommendations presented: (1) Establish a comprehensive sample library and database of amphibians and their symbiotic microbiota to create a solid foundation for scientific research. (2) Explore the coevolutionary paths between amphibians and symbiotic microbiota to clarify the dynamic evolutionary patterns and principles of their interactions. (3) Strengthen research on specific areas of amphibians, especially the microbial communities in the oral cavity and cloaca. (4) Enhance research on the symbiotic microbiota of the Gymnophiona. (5) Strengthen international cooperation to build cross-border research platforms and jointly promote the rapid development of global amphibian symbiotic microbiology. This article summarizes the current research progress on the interaction between amphibians and their symbiotic microbiota (not necessarily mutualistic). It discusses the conservation of amphibian biodiversity from the perspective of their symbiotic microbial communities and provides a forward-looking analysis of future research directions. It aims to provide rich background information for understanding the complexity of this symbiotic system, while also having significant value in enhancing the effectiveness of amphibian biodiversity conservation.

## Simple Summary

Amphibians constitute the most threatened group of vertebrates globally. Their interactions with symbiotic microorganisms are crucial for their health, reproductive efficiency, and environmental adaptability. This article synthesizes systematic assessment frameworks and methodological approaches for studying amphibian–microbiota interactions. It also explores the impact of environmental and biological factors on the characteristics of amphibian symbiotic microbial communities. Priority research trajectories should include: (1) establishing curated specimen repositories with associated metadata, (2) elucidating coevolutionary mechanisms through phylogenomic approaches, (3) intensifying investigations into niche-specific microbiota (e.g., oropharyngeal and cloacal communities), (4) prioritizing studies on Gymnophiona symbionts, and (5) fostering global collaborative networks. These efforts are of significant importance for understanding the complexity of amphibian symbiotic systems and improving the effectiveness of amphibian biodiversity conservation. These initiatives will enhance our understanding of amphibian–microbe symbiosis and improve conservation strategies for imperiled amphibian biodiversity.

## 1. Introduction

**Figure 1:**
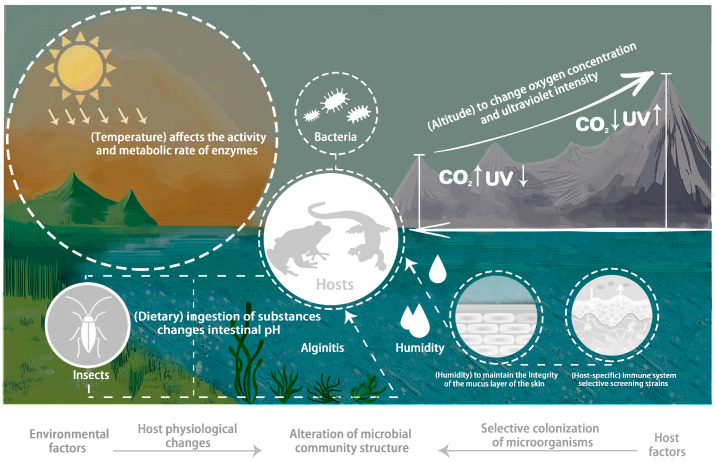
Mechanisms of environmental and host factors shaping amphibian–bacterial symbiosis.

Amphibians are characterized as metamorphosing tetrapods that lead an aquatic larval life and subsequently adopt a semi-aquatic or terrestrial lifestyle as adults, exhibiting ectothermic physiology. There is considerable diversity within this group, with some species being entirely aquatic, such as the Japanese giant salamander (*Andrias japonicus*), and others leading a fully terrestrial existence, like members of the family *Plethodontidae*, which are lungless and adapted to life on land [1]. However, some amphibians exhibit viviparous or ovoviviparous reproduction, bypassing the need to lay eggs. In these species, hatchlings emerge from the eggs having nearly completed metamorphosis, while others retain their larval form throughout their lives. The emergence of amphibians not only signifies a pivotal step in the evolution of vertebrates from aquatic to terrestrial habitats but has also given rise to more advanced vertebrates, including reptiles, birds, and mammals [2]. Amphibians hold a significant position in the evolutionary history of vertebrates [3]. With highly permeable skin and a strong dependence on environmental health, amphibians are indicators of ecological well-being and sustainable development. However, habitat loss and fragmentation, chemical pollution, pathogen infections, human activities, invasive species, and climate change have severely threatened amphibians, making them the most endangered vertebrates. Their populations are declining, with 40.7% of global amphibians and 37.05% of Chinese amphibians threatened [4]. Amphibians play an essential role in conserving biodiversity [5]. In China, there are several amphibian hotspots associated with the southeastern mountains, which are largely unprotected and under significant threat from anthropogenic pressures. These hotspots warrant greater attention in the future compared to the more well-known ones [6]. Amphibians are important components of ecosystems, and their symbiotic microbiota play a key role in maintaining ecological balance. By elucidating the mechanisms of the factors that influence these microbiota, we can better understand the ecological requirements of amphibians, thereby developing more effective conservation strategies (Figure 1). The conservation of amphibians is both imminently urgent and internationally relevant.

**Figure 2:**
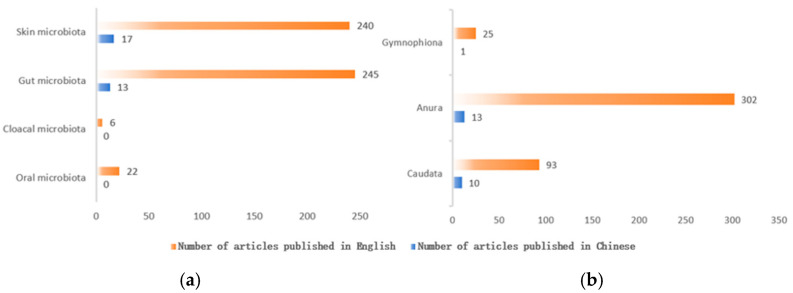
(**a**) Distribution map of research on different groups of amphibians from 2004 to 2024; (**b**) distribution map of research on symbiotic microbiota in amphibians from 2004 to 2024.

Amphibians are divided into three orders: Caudata, Anura, and Gymnophiona. Current research mainly focuses on Caudata and Anura, with relatively less on Gymnophiona (see Figure 2a for symbiotic microbiota of different amphibian groups). Given the limited research on the symbiotic microbiota of the order Gymnophiona, we will primarily focus on the orders Caudata and Anura. However, it is important to recognize that research on the symbiotic microbiota of Gymnophiona is also crucial, and efforts should be increased in this area in the future. Gymnophiona, a distinctive order of amphibians renowned for their worm-like bodies, lack of limbs, and significantly reduced or absent eyes [7], predominantly inhabit moist terrestrial or aquatic environments, emerging nocturnally to feed on insects and worms. Their reproductive strategies encompass both oviparity, where eggs are laid, and viviparity, characterized by the birth of live young. Notably, certain species exhibit filial cannibalism, with larvae consuming nutrients secreted by the female prior to hatching. Gymnophiona are widely distributed across tropical regions, particularly in equatorial forests and savannas. However, due to their secretive lifestyle and the resultant difficulties in observing them in their natural habitat, comprehensive studies on their symbiotic microorganisms remain scarce. This gap in knowledge is significant as Gymnophiona play crucial roles in ecosystem dynamics and offer unique insights into the evolutionary history and biodiversity of amphibians, highlighting the need for further investigation into this enigmatic group.

The skin, gut, oral cavity, and cloaca of vertebrates are colonized by trillions of microbial cells, including bacteria, fungi, algae, and others. Few studies of host-associated microbiomes focus on fungi. This is because it is difficult to discern whether a fungal organism found on amphibians is associating with them in a durable, symbiotic interaction versus a transient one, and to what extent the habitat and host share microbes. Although some studies have investigated fungal taxa in amphibians, these communities are likely to vary seasonally and among individuals, making it challenging to specifically address these changes [8]. The algal microbiome is highly dynamic, with its composition and function varying significantly with seasons, environmental conditions, and host status. This dynamism increases the complexity of research, making long-term and systematic studies more challenging [9].Therefore, this article will primarily discuss bacteria. Specific bacterial species occupy distinct ecological niches, contributing to the host’s immune system, physiological processes, and long-term interactions that foster adaptive evolution. This entire microbial ecosystem is referred to as the symbiotic microbiome [10]. The symbiotic microbial ecology is shaped by complex dynamic interactions influenced by both exogenous and endogenous factors, offering rich research avenues in ecology, physiology, pathology, metabolism, and evolution. Assessing the composition and function of symbiotic microbiota has recently become technically feasible and cost effective [11]. For example, over the past decade, metagenomic analysis has provided valuable insights and tools for identifying microbial communities and their functions. Single-cell transcriptomics technology can reveal the gene expression of microorganisms within the host, providing deeper insights into the interactions between microorganisms and their hosts. It also helps to uncover the complexity and functional diversity of microbial communities [12]. Existing research mainly focuses on mammalian hosts, while studies on the coevolutionary relationships between amphibian hosts and their symbiotic microbiota are relatively scarce. Thus, enhancing research on amphibians is urgent, as it may guide decisions and management approaches for amphibian conservation from a microbiota perspective.

The impact of symbiotic microbiota on amphibian hosts is likely profound and closely related to the long-term health prospects of the host. Current research on symbiotic microbiota primarily focuses on skin and gut microbiota. Symbiotic microbiota plays a crucial role in the adaptability of wild amphibians, and identifying factors that influence symbiotic microbiota will open new avenues for improving host health and monitoring their nutritional status.

**Figure 3:**
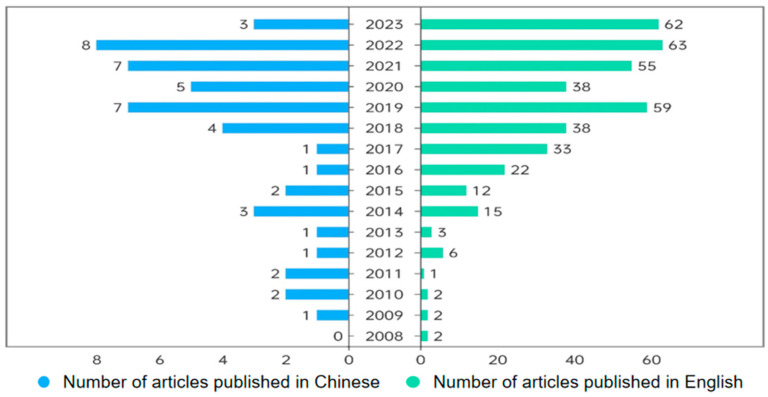
Number of articles on amphibian symbiotic microbiota from 2004 to 2024.

Studies on the symbiotic microbiota of amphibians have yielded significant findings both domestically and internationally. The literature in different languages reflects varying research contexts and cultural backgrounds. By separately searching Chinese and English databases, researchers can gain insights into the trends and focal points in each linguistic environment. This approach facilitates the identification of cross-cultural research disparities and connections. Additionally, conducting separate searches helps researchers efficiently locate relevant information, thereby enhancing the efficiency and quality of their studies. In the Web of Science (http://apps.webofknowledge.com) (accessed on 10 March 2024) database and Scopus (https://www.scopus.com) (accessed on 10 March 2024), the literature published up to 2024 regarding the symbiotic microbiota of amphibians was searched using the terms “Symbiotic microbiota” or “Skin microbiota” or “Gut microbiota” and “Amphibian”, with publication years limited to 2004–2024 and document type set to “Article”. Articles that are not representative of the research field were excluded after reviewing abstracts and keywords. Chinese literature was retrieved from China National Knowledge Infrastructure (https://www.cnki.net) (accessed on 10 March 2024), applying identical criteria. A total of 487 articles were collected, including 428 in English and 59 in Chinese. Through a comprehensive review of the literature published between 2004 and 2024, it is evident that the number of publications focused on amphibians and their associated microbiota has shown a steady increase over the years (Figure 3). Over the past decade, with the widespread application of next-generation sequencing technologies, there has been growing attention to the composition and function of amphibian symbiotic microbiota and the interactions between hosts and their symbiotic microbiota.

**Figure 4:**
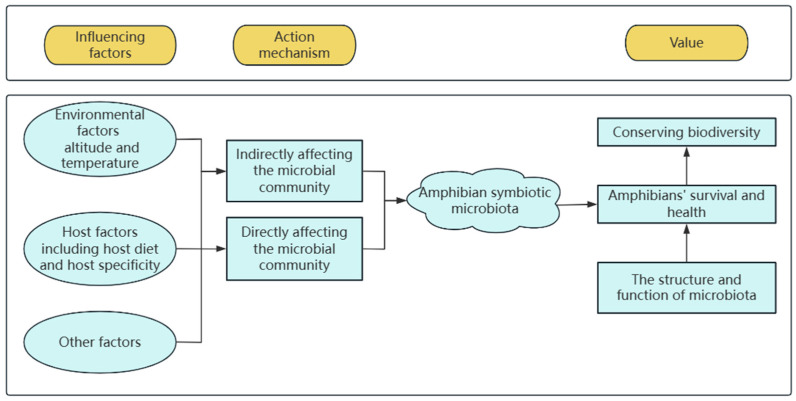
Outline of the research framework for symbiotic microbiota in amphibians.

This article summarizes the research progress of amphibian symbiotic microbiota in recent years, focusing on the research progress of genomics technology and the tandem synergistic effect of amphibian symbiotic microbiota and environment, in order to emphasize the role of microbiota in the protection of biodiversity and to look forward to future research trends. It provides a background reference for research on amphibian symbiotic microbiota (see Figure 4 for the research framework of amphibian symbiotic microbiota).

## 2. The Importance of Amphibian Symbiotic Microbiota

Symbiotic microbiota play crucial roles in host immunity [13,14], adaptation to extreme ecological niches [15], and the transmission of chemical information [16]. These microbiota are widely distributed across various parts of the host’s body, with existing studies primarily focusing on the oral, intestinal, skin, and cloacal microbiota (see Figure 2b for an overview of studies on different amphibian symbiotic microbiota). The oral microbiota serves as a bridge between the external environment and the host’s digestive tract, influencing pathogen defense and nutrient absorption [17]. The gut microbiota of amphibians is essential for maintaining host health and facilitating evolutionary adaptation [18]. The skin microbiota is particularly significant in disease resistance [13,14,19]. Meanwhile, the cloacal microbiota helps hosts resist pathogenic invasions through competitive exclusion or the production of antimicrobial substances, thereby maintaining ecological balance and reducing infection risks [20]. Investigating the symbiotic microbiota of amphibians can provide valuable insights into how hosts adapt to environmental changes, including the impact of altitude variations on symbiotic microbes. This research is of significant importance for predicting and addressing the effects of global climate change on biodiversity. A considerable body of evidence indicates that studying the dynamics of symbiotic microbial composition and diversity is beneficial for understanding the immune and health status of hosts [21]. Despite the extensive distribution of symbiotic microbiota, current research has predominantly focused on gut and skin microbiota. Therefore, this article delves into these two domains in greater depth.

### 2.1. The Importance of Amphibian Gut Microbiota

The gut microbiota represents a complex ecosystem that is prominently featured among symbiotic microbiota and is predominantly composed of bacteria in terms of quantity [22]. The composition of the gut microbiota is unique to each individual and is influenced by multiple factors, including host gut homeostasis and metabolism [23,24]. This microbiota plays a vital role in the physiology and health of the host [25], contributing to various physiological processes such as nutrient fermentation, intake, digestion, metabolism, and immune responses. Moreover, the gut microbiota is intricately connected to other organs, such as the liver and brain, forming a gut–liver–brain axis that influences overall host health [26]. The coevolution between the host and its gut microbiota facilitates the adaptation of organisms to their specific ecological niches [27]. In recent years, the role of gut microbiota in biodiversity conservation has garnered increasing attention. Understanding the various factors that influence the assembly and structure of host gut microbial communities, clarifying the dynamic changes in the composition and diversity of host gut microbiota, and elucidating the adaptive mechanisms developed by host gut microbiota in response to various external disturbances are all significant for the conservation of amphibians [28]. These insights can provide valuable guidance for predicting and mitigating the impacts of environmental changes on amphibian populations.

### 2.2. The Importance of Amphibian Skin Microbiota

The skin of amphibians is an extremely complex organ involved in respiration, osmoregulation, temperature regulation, pigmentation, chemical communication, and pathogen defense [26]. It enhances barrier protection through the synergistic effects of physical (mucus and stratum corneum), chemical (antimicrobial peptides and toxins), and immune cell characteristics. The mucosal layer is rich in glycoproteins, microbial symbionts, and pathogens [27]. Specific oligosaccharides mediate microbial interactions throughout all life stages of amphibians, from eggs to adults [28,29].

The skin is colonized by symbiotic microbiota that inhabit the body surface, acting as a barrier against invasion, colonization, and infection by foreign pathogenic microbiota [30]. This microbiota, primarily composed of bacteria, fungi, and viruses, drives metabolism and immunity, playing a key role in maintaining amphibian health and survival [31]. The microbial community, which serves as a natural immune barrier, is closely related to the skin and engages in complex interactions with the host. These interactions stimulate a range of innate immune responses, enhancing the complexity and stability of the skin microbiota [32]. The formation and enhancement of skin barrier characteristics are dynamically influenced by microbial communities, which integrate with environmental microbial factors to establish an interactive mechanism that facilitates stable microbial colonization. This dynamic equilibrium is crucial for maintaining skin health and defending against external pathogens.

## 3. Research Progress of Microbiological Classification Technology

The development of bacterial classification and identification methods has progressed from morphological observation to molecular biology, encompassing several key stages.

In 1676, Antonie van Leeuwenhoek observed bacteria using his homemade microscope, marking the beginning of microbiology. During this initial phase, bacterial classification relied primarily on morphological characteristics such as shape, size, and staining reactions.

After 1923, advancements in microbiology introduced more classical indicators for bacterial identification, including morphology, physiological and biochemical properties, ecological habits, life history, and serological reactions. These methods became the mainstream for bacterial classification and identification at the time.

In the 1960s, technological progress led microbiologists to adopt physical and chemical analysis methods and develop numerous automated instruments. These innovations accelerated and simplified bacterial identification, achieving automation and mechanization. For example, the development of microscale rapid media and biochemical reaction systems significantly improved identification efficiency. The advent of electron microscopy and computer technology, along with DNA-DNA hybridization techniques, shifted bacterial classification from morphological physiology to molecular biology. Additionally, advances in sequencing technology enabled the decoding and documentation of microbial genomes, facilitating modern classification based on phylogenetics.

During the 1960s and 1970s, scientists began analyzing and comparing the structural features of biomacromolecules, particularly proteins, RNA, and DNA. The 16S rRNA gene emerged as an excellent “molecular ruler” for phylogenetic analysis, significantly advancing bacterial taxonomy [33]. As a highly conserved molecular marker in evolutionary processes, the 16S rRNA gene sequence exhibits sufficient variation between species while maintaining relative consistency within species, making it an effective tool for distinguishing and identifying microbial species [34]. The advent of biotechniques based on 16S rRNA analysis has significantly expanded the scope of microbial research. Currently, microbial groups are primarily identified through 16S rRNA gene sequence analysis, which involves DNA extraction, PCR amplification, library construction, sequencing, and sequence analysis [35]. By comparing and constructing phylogenetic trees from 16S rRNA gene sequences of different microbes, their evolutionary relationships can be revealed, aiding in understanding microbial diversity and population evolution. Without relying on cultivation, total DNA can be directly extracted from environmental samples to analyze the structure of microbial communities by sequencing 16S rRNA gene fragments, making it suitable for studying microbial diversity in complex ecosystems.

In summary, the development history of bacterial classification and identification methods reflects continuous progress and improvement. Each technological innovation has significantly advanced the field of microbiology. From initial morphological observations to current molecular biological techniques, these advancements have deepened our understanding of bacterial diversity and provided important tools for disease diagnosis and treatment.

## 4. Factors Affecting Amphibian Symbiotic Microbiota

Understanding how host development and environmental variables influence the microbiome is a central goal in studying microbial communities in amphibians and other host taxa [36]. Factors affecting amphibian symbiotic microbiota are diverse and complex, encompassing environmental conditions (e.g., temperature, pH, and humidity), host factors (e.g., species, gender, and life stages), and human activities. These factors interact to shape the dynamic changes in amphibian symbiotic microbiota. To protect amphibian health and biodiversity, comprehensive measures are needed to mitigate negative impacts. Focusing on these factors elucidates the dynamic relationship between amphibians and their microbiota, promoting harmonious coexistence. This section will discuss the specific effects of temperature, altitude, humidity, host diet, and host specificity on amphibian symbiotic microbiota, aiming to uncover the causes of amphibian diseases and promote species diversity protection.

### 4.1. Environmental Factors

The environment can directly influence microbial communities by affecting the affinity of microbiota for substrates, thus impacting their growth. For instance, under certain conditions, elevating temperature may foster the growth of beneficial microbes while suppressing the proliferation of potentially harmful ones. Such alterations could either enhance or diminish the amphibians’ capacity to resist *Batrachochytrium dendrobatidis* (Bd) infections [37]. This is attributable to the fact that different microbes have distinct optimal temperature ranges for growth; when environmental conditions fluctuate, those microbes adapted to the new conditions gain a competitive edge. The environment can also indirectly affect bacterial communities by altering the chemical composition of amphibian skin [38], regulating the growth of certain microbes. Changes in pH, for instance, can influence the growth of pH-sensitive microbiota by promoting the proliferation of acidophilic or alkaliphilic microbes, or inhibiting those that do not tolerate extreme pH values. Amphibian skin is a special environment that specifically hosts major bacterial taxa (such as *Proteobacteria*, *Bacillota*, and *Bacteroidetes*) and fungi [39,40]. Due to direct contact with the environment, skin microbiota are more susceptible to environmental factors such as temperature and humidity [41,42]. Additionally, the gut microbiota of frogs varies across different habitats, aiding hosts in more effectively utilizing food resources to adapt to environmental changes [43,44].

#### 4.1.1. The Impact of Temperature on Amphibian Symbiotic Microbiota

Throughout their evolutionary history, amphibians have faced an unstable global climate. Anthropogenic climate change can cause long-term warming of aquatic environments, impacting amphibian survival [45]. Temperature affects the composition of amphibian symbiotic microbiota, as different microbiota types have varying temperature sensitivities. Heat-tolerant or cold-tolerant microbiota may dominate under specific temperatures, while temperature-sensitive microbiota may decline [46]. Amphibian skin and gut microbiota show seasonal similarities, with the gut acting as a reservoir for skin bacteria that can be re-inoculated via the cloaca [47]. Gut microbiota can also be transmitted to the soil through defecation and from the environment back to the skin [48,49]. Frogs often use their front legs to push prey into their mouths, transferring environmental bacteria from the surroundings into the oral cavity and then the gut [19]. Most amphibians practice dermatophagy, consuming their shed skin, which likely cycles microbiota between the skin and gut [40,50]. Fasting or cessation of defecation may halt the exchange between gut and skin microbiota, potentially explaining seasonal variations in their similarity [19].

##### The Impact of Temperature on Amphibian Gut Microbiota

The composition of animal gut microbiota largely depends on the external environment they inhabit, and the impact of temperature on the amphibian gut microbiota has increasingly attracted attention in recent years. Temperature can directly alter the gut microbiota, especially in ectothermic animals that cannot precisely regulate their body temperature [51].

Studies on Caudata have shown that temperature is a key factor influencing gut microbiota community structure. Fontaine et al. [37] collected samples of the gray-backed salamander (*Plethodon cinereus*) in Pembroke, Virginia, USA, and compared the diversity and composition of the salamander gut microbiota kept at experimental temperatures of 10 °C, 15 °C, and 20 °C. They found that the diversity of salamander gut microbiota decreased at 20 °C, indicating that higher temperatures significantly reduce gut microbiota diversity. Community composition also varied at different environmental temperatures. This demonstrates that temperature can affect the diversity of amphibian gut microbiota, with different temperature gradients yielding different results. The significant reduction in gut microbiota diversity at high temperatures is primarily due to the impact of high temperatures on microbial physiological functions and survival. Understanding microbial responses to temperature helps predict the impact of global climate change on ecosystems, particularly those that rely on specific microbiota, such as coral reefs [52], wetlands [53], and agricultural soils [54].

In studies on Anura, the diversity and richness of gut microbiota are influenced by temperature. Ren et al. [45] exposed the tadpoles of Chinese brown frog (*Rana chinensis*) to different thermal groups, collected intestinal samples from twelve temperatures, and studied the variations in gut microbiota by Illumina sequencing. The results showed that exposing these tadpoles to different temperatures led to significant changes in gut microbiota composition, with increased diversity and richness in the gut microbiota community in the high-temperature group compared to the control group. However, Li obtained somewhat different conclusions in their study, which examined changes in the gut microbiota of the African clawed frog (*Xenopus tropicalis*) under controlled laboratory conditions at different temperatures [55]. Their results indicated that the composition of the gut microbiota was comparable between warm and cold temperatures. Temperature affected the β-diversity of the gut microbiota but did not influence α-diversity. These two studies reached different conclusions potentially due to the temperature settings. Different temperature gradient setups led to different results. Tong collected *R. dybowskii* from Luobei County, Heilongjiang Province, China, at different temperatures and studied their gut microbiota using Illumina sequencing [19]. Their study demonstrated that environmental temperature alters the amphibian gut microbiota. However, the mechanisms by which gut microbiota respond to temperature feedback, and how these responses affect host phenotype and adaptability, remain to be resolved. Future research might consider the metabolic pathways within the microbiota community, as well as patterns of microbial gene expression and epigenetic states.

##### The Impact of Temperature on Amphibian Skin Microbiota

Exposure to different climates can alter the structure of skin microbiota, which is particularly relevant for ectothermic animals like amphibians [56]. Seasonal changes can also influence host behavior by increasing body temperature, which in turn may alter the structure of skin microbiota [57]. Higher temperatures increase the frequency of skin shedding, thereby reducing bacterial abundance on the skin through frequent disturbances [58,59]. Hot conditions can affect the activity of antifungal metabolites produced by amphibian skin symbiotic microbiota [60], while lower temperatures may limit the production of antimicrobial metabolites by probiotics [61]. Current research provides limited understanding of the comprehensive impact of environmental changes on microbiota stability, and these impacts may vary among species from different habitats and ecosystems. Future investigations into the seasonal variations and long-term trends of microbiota under different environmental conditions will enable the exploration of microbiota response patterns to both short-term and long-term environmental changes. This, in turn, will lead to a deeper understanding of the adaptive strategies that microbes employ in response to environmental alterations.

Studies on Caudata have shown that temperature can lead to increases or decreases in the abundance of certain bacterial species [19]. In studies on the gray-backed salamander, bacteria resistant to chytrid fungus (*Batrachochytrium dendrobatidis*, Bd) may gain a competitive advantage at higher temperatures, as they are more likely to produce inhibitory metabolites. As temperatures rise, the abundance of OTUs resistant to Bd increases, which may indirectly reduce skin microbiota α-diversity. Temperature may also indirectly affect microbiota structure through host physiological changes, such as immune function. For example, during winter, many amphibians enter hibernation to cope with cold environments, which results in a significant drop in body temperature to conserve energy. This decrease in body temperature leads to changes in the host’s immune system and slows down the metabolic rate of skin microbiota [62]. These findings demonstrate that temperature does indeed influence the composition and richness of certain microbiota. Temperature directly influences the structure of amphibian skin microbiota by affecting bacterial communities. Additionally, it indirectly impacts the host’s physiological status, thereby significantly altering the skin microbiota community structure across different seasons and environmental conditions. These findings are crucial for understanding how amphibians cope with environmental changes and how to protect them from pathogens.

In studies on Anura, temperature affects the composition of skin microbiota. From summer to winter, the α diversity of the skin microbiota significantly decreases. Furthermore, there are significant differences in the composition and structure of the skin microbiota between these two seasons [63]. While this study emphasizes the role of temperature as a critical environmental factor in regulating the amphibian skin microbiota, it also cautions us to approach experimental conclusions with prudence, giving due consideration to the complexity of the winter niche. For example, hibernation behaviors and reduced food intake are unique to winter and may also have a non-negligible impact on the skin microbiota. To better understand the effects of temperature on amphibian skin microbiota, future research should adopt multifactorial analysis. This approach should consider the behavioral habits and physiological states of animals across different seasons. By doing so, researchers can separate the influence of temperature from other variables. This will enable accurate deciphering of how temperature acts alone or in conjunction with other factors. Ultimately, this research will help shape the diversity and stability of the amphibian skin microbiota.

Collectively, while empirical evidence substantiates the pivotal role of temperature in modulating skin microbiota composition, comprehensive elucidation of the underlying mechanisms necessitates additional inquiry. Such endeavors will demand more sophisticated experimental paradigms, especially those capable of disentangling the complex interplay of multiple environmental determinants across seasonal transitions. This not only deepens understanding of skin microbiota ecosystems but also provides a scientific basis for developing strategies for the conservation and health management of animals in specific ecological environments.

#### 4.1.2. The Impact of Altitude on Amphibian Symbiotic Microbiota

Maintaining oxygen homeostasis in the gut is crucial for sustaining healthy gut microbiota. However, few studies have examined how atmospheric oxygen concentration influences the gut microbiota of natural populations. High-altitude environments offer unique opportunities to explore the potential impacts of atmospheric oxygen on the composition and function of gut microbiota.

Altitude is a complex gradient where many environmental variables, such as temperature, humidity, and ultraviolet intensity, change. When discussing the impact of elevation on amphibian symbiotic microbiota, we must consider not only elevation but also related environmental factors. Studies have shown that microbial community structures vary with altitude [64,65]. Altitude can significantly affect the composition and structure of amphibian symbiotic microbiota, with a more pronounced impact on skin microbiota than on gut microbiota [66].

##### The Impact of Altitude on Amphibian Gut Microbiota

At high altitudes, oxygen levels are lower, which may affect the growth and metabolic activities of amphibian gut microbiota [66]. Some studies have shown that the diversity of amphibian gut microbiota living in low-oxygen environments is lower. The availability of food sources at high altitudes may also differ from those at lower altitudes, which is another factor influencing amphibian gut microbiota [66].

Research exploring the impact of altitude on the gut microbiota of Caudata is still in its infancy and lacks systematic summaries. Although research on Caudata is relatively scarce, studies on Anura have yielded more mature results. In studies on Anura, significant differences exist between gut microbiota at different altitudes. Xu LL collected 67 gut samples in Jiangle Town, Leshan City, Sichuan Province, including samples from five species (*Bufo gargarizans*, *Microhyla fissipes*, *Pelophylax nigromaculata*, *Rana omeimontis*, and *Fejervarya limnocharis*) at low altitude and five species (*Bufo gargarizans*, *Scutiger boulengeri*, *Batrachuperus tibetanus*, *Amolops xinduqiao*, and *Nanorana pleskei*) at high altitude [66]. After 16S rRNA gene sequencing and analysis of the data’s diversity, the results showed that a higher proportion of *Bacillota* and a lower proportion of *Bacteroidetes* in the gut microbiota were better suited for high-altitude environments. Moreover, a higher proportion of *Ruminococcaceae* and *Christensenellaceae* could help hosts survive in high-altitude environments [67]. Other studies have found that the gut microbiota of mammals living at high altitudes can help the host adapt to high-altitude environments by increasing digestive efficiency [68]. Results revealed significant differences in the number of observed OTUs between gut microbiota at high and low altitudes.

During studies on the impact of altitude changes on the amphibian gut microbiota, an interesting phenomenon was observed: as altitude gradually increases, the microbial diversity in some animals’ guts decreases significantly. This phenomenon indicates that high-altitude environments may pose challenges to the stability of amphibian gut ecosystems, which provides new directions and deeper insights for future research.

##### The Impact of Altitude on Amphibian Skin Microbiota

The composition of substances in the environment affects the skin microbiota. As altitude changes, factors such as temperature, humidity, and ultraviolet radiation intensity also change, which may affect the structure and function of the skin microbiota by influencing the composition of substances in the host and the environment. Environmental samples (such as water and soil) at different altitudes may also impact the amphibian skin microbiota. Although skin microbiota have been shown to play important roles in the health and disease of several species [69], the impact of altitude on the skin microbiota and the relationship between high-altitude skin microbiota and health and disease states remain largely unknown. To explore the connection between the skin microbiota in high-altitude regions and individual health and disease status, a feasible approach is to focus on the unique environmental conditions of these regions. Specifically, detailed examination of factors such as oxygen scarcity, ultraviolet radiation intensity, temperature fluctuations, and air humidity can reveal how these factors collectively impact the microbial ecosystem on the skin surface. A systematic investigation can elucidate whether specific environmental pressures induce alterations in the structural and functional composition of the skin microbiota. Additionally, potential associations between these microbial shifts and various skin health conditions can be evaluated, including changes in disease susceptibility and skin’s adaptive capacity. This series of studies not only enriches the understanding of the dynamics of the skin microbiome in high-altitude environments but also helps elucidate its role in maintaining skin health and the mechanisms involved in disease induction.

Like mammals, the amphibian skin microbiota may also be influenced by altitude-related environmental factors such as changes in temperature, humidity, and ultraviolet radiation intensity [70]. Therefore, exploring the altitudinal distribution of amphibian skin microbiota can deepen understanding of microbial ecology and reveal potential mechanisms by which these animals adapt to high-altitude environments.

In studies on Caudata, the impact of different altitudes on amphibian skin microbiota varies. Muletz Wolz sampled the skin microbiota of three coexisting lungless salamanders (*Plethodon cinereus*, *Plethodon glutinosus*, and *Plethodon cylindraceus*) along an altitudinal gradient in the central Appalachian Mountains [71]. They determined that altitude can affect the structure of the skin microbiota, with α diversity increasing with altitude and the abundance of 17 bacterial OTUs changing with altitude (16 OTUs decreasing and 1 OTU increasing), which confirmed that altitude influences the composition of amphibian skin microbiota. However, this contrasts with the findings of Zeng et al. [72], who observed a decrease in α-diversity with increasing altitude. The differences in α-diversity between the two studies may primarily be due to differences in species and environments. Wolz et al. sampled the skin of lungless salamanders at altitudes ranging from 700 to 1000 m, which Zeng’s study considered low altitude, while their high-altitude environment ranged from 3750 to 3861 m.

In Anura, altitude also affects the composition and richness of amphibian skin microbiota. Xu LL conducted experiments on the impact of altitude on amphibian skin microbiota [66]. The results showed that at the phylum level, the skin microbiota at high and low altitudes are largely similar, primarily consisting of *Proteobacteria*, *Bacteroidetes*, and *Actinomycetota*. However, *Chloroflexi* and *Planctomycetes* were only detected at high altitudes, indicating that altitude affects the composition of amphibian skin microbiota. Statistical analyses revealed significant differences in the number of observed OTUs between the skins at high and low altitudes. It was also found that the number of OTUs on the skin was significantly higher than that in the gut within the same altitude environment. The similarity between skin microbiota of high-altitude species was significantly higher than that of low-altitude species, but no such pattern was observed in gut microbiota. This finding may further support the selective pressure of high-altitude environments on skin microbiota communities. It was also discovered that the skin microbiota at high altitudes had a higher proportion of biofilm formation, which was not observed in the gut microbiota at high altitudes. This could be because skin microbiota may rely more on biofilm formation to resist external stress, while gut microbiota may maintain homeostasis through other mechanisms.

Some studies have combined metagenomics, transcriptomics, and ecological models to reveal the assembly patterns of amphibian symbiotic microbial communities across different altitudinal gradients. The composition and diversity of symbionts show variability across altitudinal gradients, and significant differences exist between skin and gut microbial communities. This indicates that these symbiotic groups play different roles in host adaptation to different altitudinal habitats. It is further suggested that habitat heterogeneity across altitudinal gradients or heterogeneity in host selection plays an important role in shaping distinct symbiotic microbial communities. Additionally, a significant correlation was observed between the differences in skin and gut microbial community structures and their community assembly processes, indicating that changes in community assembly processes can shape distinct symbiotic microbial communities [73].

Studying the impact of altitude on amphibian symbiotic microbiota not only helps us better understand the adaptive mechanisms of organisms in high-altitude environments but also provides important references for ecological conservation and biodiversity research.

#### 4.1.3. The Impact of Host Diet Composition on Amphibian Symbiotic Microbiota

Host diet significantly influences the abundance, composition, and functional capacity of the gut microbiota [74]. Fasting can markedly alter the host’s gut nutritional status, thereby changing the density, composition, and metabolic capabilities of the microbiota [75]. The type of food consumed directly affects the nutritional supply for gut microbiota. For instance, the Chinese giant salamander (*Andrias davidianus*) exhibits changes in its gut microbiota as it grows [76]. As the salamander develops from a tadpole to an adult, its diet shifts from aquatic invertebrates to a more diverse range of prey, including fish and other larger organisms. This dietary transition significantly impacts the composition of the gut microbiota, which in turn affects the health and physiological functions of the salamander.

##### The Impact of Host Diet Composition on Amphibian Gut Microbiota

Although knowledge of the environmental impacts on the amphibian gut microbiota is continuously expanding, our understanding of the composition, source, and dynamics of the gut microbiota due to external environments remains limited. Supplementing this understanding will help maintain the health of amphibians, which is essential for the conservation and management of amphibian gut microbiota. Dietary habits contribute to the selection and reshaping of the host’s gut microbiota [77]. Dietary habits, nutritional content, and food diversity can influence the composition of the amphibian gut microbiota. Captivity can improve the organization of the gut tissue, affect the structure of the gut microbiota, and increase the abundance of beneficial bacteria to enhance gut immune function [78]. However, previous research has paid less attention to the impact of diet on the diversity of the amphibian gut microbiota, and it is unclear how quickly the composition of the gut microbiota changes with dietary changes.

In studies on Caudata, the appearance of the gut microbiota as a key regulator of health and disease complicates this issue. There is a reciprocal relationship between diet and the gut microbiota, making dietary factors one of the most effective regulators of microbiota composition and function [79]. The gut microbiota, in turn, affects the absorption, metabolism, and storage of ingested nutrients, potentially having far-reaching effects on host physiology. Studies have compared the gut microbiota of the Oriental newt (*Cynops orientalis*) under different dietary conditions, finding that the gut microbiota richness and diversity were higher in the wild group than in the captive group [80]. These findings suggest that dietary habits can regulate gut microbiota structure and alter microbial ecosystem function. Rich natural diets may enhance gut microbiota diversity in wild Oriental newts, improving functional capacity and microbial community resilience to disturbances [81].

In studies on Anura, the host’s diet composition directly influences the structure and function of the gut microbiota. Sarah A. Knutie et al. fed Cuban treefrog (*Osteopilus septentrionalis*) tadpoles two different diets and found that diet affected the bacterial community in tadpole guts but did not have lasting effects on adult bacterial communities [82]. Conversely, tadpole diet had lasting effects on resistance and tolerance to infection in adult frogs. The diet of Cuban treefrog tadpoles influenced their gut microbiota and defense against intestinal nematodes, but these effects depended on the host and infection stage. In 2018, Demircan et al. found that diet and host epigenetics had separate impacts, with diet strongly influencing gut bacterial diversity while host epigenetics exerted a greater selective effect in gut tissues [83]. These findings underscore the importance of understanding the complex interactions between diet, microbiota, and host health. Future research might adjust diets to promote beneficial bacterial growth, thereby optimizing microbiota structure and enhancing host health.

The structure of the gut microbial community is determined by the type of food ingested. Different foods provide various nutrient sources that directly affect the gut environment. These food sources have different nutrient compositions that may lead to the growth or decline of certain microbial populations. Therefore, differences in diet components can cause changes in the diversity and function of the gut microbiota, which may further affect the health status of amphibians and their ability to adapt to their environment. For amphibians, the gut microbiota plays a critical role in digestion and nutrient absorption, which is particularly important when they face frequent environmental changes and transitions in their life stages [84].

##### The Impact of Host Diet Composition on Amphibian Skin Microbiota

The conditions in which amphibians are kept, such as diet, substrate, and enrichment, can potentially affect the composition of bacterial communities, with significant differences observed between wild and captive host diets.

In studies on Caudata, Sabino-Pinto et al. compared the skin bacterial communities of 61 amphibian species (both wild and captive) from Hiroshima, Japan, using high-throughput amplicon sequencing of the 16S rRNA gene fragment [85]. They found significant differences in skin bacterial community structure between wild and captive red-bellied newts (*Cynops pyrrhogaster*), with higher α-diversity in wild individuals. The core bacterial community in captive red-bellied newts was dominated by Xanthomonadaceae (23.9%) and Pseudomonadaceae (18.9%), while in wild populations, it was dominated by Comamonadaceae (44.9%) and Verrucomicrobiaceae (19.2%). Although wild individuals had more diverse communities and greater phylogenetic diversity, their OTU richness was similar to that of captive individuals. Hernández-Gómez et al. collected skin microbiota from wild and captive hellbenders (*Cryptobranchus alleganiensis*) from two subspecies in eastern Missouri and the Ozark region [86]. They found that in the Ozark region, wild individuals had higher skin bacterial diversity than captive ones, while in eastern Missouri, captive individuals had higher diversity. Significant differences were also noted between wild and captive individuals in microbial community composition and predicted metagenomes, as well as in the abundance of Proteobacteria, Bacteroidetes, and Verrucomicrobia. These results suggest that captive hellbenders have different microbial communities than their wild counterparts, with wild hellbenders’ skin microbiota containing more metabolism-related genes, indicating higher metabolic plasticity. The contrasting results from the two regions may be due to uncontrolled factors in the wild and captive environments, such as temperature and host specificity, which make consistency challenging. Despite differing outcomes, both studies confirmed that captive environments impact the composition and richness of amphibian skin microbiota.

In Anuran research, Rachael E. Antwis successfully demonstrated that the availability of carotenoids affects the overall community composition, species richness, and abundance of bacteria associated with the skin of captive frogs [48]. Compared to those fed a carotenoid-free diet, red-eyed tree frogs (*Agalychnis callidryas*) fed a carotenoid-rich diet had greater species richness and bacterial abundance. This suggests that the availability of carotenoids in the diet of captive frogs may benefit skin-associated bacterial communities. It was also found that wild red-eyed tree frogs harbored more than twice the number of different bacterial species compared to captive populations, with little overlap between species. This indicates that captive populations may support reduced and differentiated bacterial communities compared to their wild counterparts, which is particularly relevant for translocation conservation projects. The bacterial community composition of frogs fed a carotenoid-rich diet was significantly different from those fed a carotenoid-free diet. Providing dietary carotenoids to captive frogs led to a significant increase in the number of different bacterial isolates on individual frogs, along with a significant increase in bacterial abundance. Compared to frogs on a carotenoid-free diet, those on a carotenoid diet had increased bacterial richness on their skin, suggesting that the presence of carotenoids can improve the growth or survival of bacteria on the skin. The impact of dietary carotenoids on the bacterial communities on frog skin could be significant for the health and immune system of frogs, as skin bacterial communities play a crucial role in preventing pathogen invasion and maintaining skin health. Managing and improving wildlife microbial communities through dietary intervention could have profound implications for animal conservation, ecological balance, and human health. Furthermore, Wilson et al. characterized microbial communities using 16S rRNA amplicon sequencing methods [87]. The analysis showed that captive individuals had significantly lower bacterial species diversity and relative abundance compared to wild populations. This could lead to captive golden mantella frogs (*Mantella aurantiaca*) being more susceptible to infection after release into the wild due to insufficient skin microbiota. It was also found that captive golden mantella frogs had significantly different skin microbial community compositions compared to their wild conspecifics. The skin bacterial communities of both captive and wild golden mantella frogs were dominated by Gammaproteobacteria and Actinomycetota, consistent with studies on amphibians in North America [88,89], Central America [39], Europe [90], and Japan [85].

In natural environments, amphibian skin microbiota is influenced by various factors, including geographical location, climate, water quality, soil type, and interactions with other organisms, among which diet plays a significant role. In contrast, the skin microbiota of captive amphibians exists in a relatively uniform and stable environment, leading to the skin microbiota of wild amphibians generally being richer and more diverse than under captive conditions.

In summary, research on amphibian symbiotic microbiota in wild and captive environments is crucial not only for the conservation and management of wildlife but also for advancing disciplines such as ecology, conservation biology, environmental science, and microbiology. These investigations may facilitate a comprehensive understanding of the health dynamics of amphibians within their indigenous environments and enable robust assessments of ecological integrity and species richness. Furthermore, they aid in the mitigation and control of amphibian disease outbreaks. Additionally, these studies can provide new insights and resources for human medicine and biotechnology, promote research on the impact of global climate change on biodiversity and ecosystem services, and improve the welfare and breeding success rates of captive animals, having far-reaching significance across multiple fields.

#### 4.1.4. The Impact of Humidity on Amphibian Symbiotic Microbiota

Humidity, as a key environmental factor, significantly influences the composition, diversity, and functional characteristics of amphibian symbiotic microbial communities through direct alteration of microbial habitats and indirect regulation of host physiological states [91].

In terms of skin microbiota, changes in humidity can markedly affect the ecological balance of microbial communities. Low-humidity environments may lead to dehydration of amphibian skin, compromising the integrity of the stratum corneum and reducing skin barrier function, thereby increasing the risk of infection by pathogenic microorganisms such as the chytrid fungus *Batrachochytrium dendrobatidis*. Research indicates that skin desiccation can reduce the diversity of symbiotic microorganisms, particularly those probiotics (such as *Pseudomonas* and *Acinetobacter* spp.) that rely on moist environments and play crucial roles in maintaining skin immune homeostasis and resisting pathogens. Conversely, high-humidity environments may provide favorable conditions for the growth of certain opportunistic pathogens (such as *Aeromonas* and *Fusarium* spp.), leading to microbial community dysbiosis and increased incidence of fungal infections and dermatological diseases. For instance, studies on the yellow-bellied toad (*Bufo gargarizans*) have shown that water humidity, temperature, and dissolved oxygen levels are significantly correlated with the diversity and composition of skin microbial communities, with humidity changes directly affecting the stability of the skin surface microenvironment [92]. Moreover, research on the northeastern wood frog (*Rana dybowskii*) further confirms that humidity indirectly influences microbial community dynamics by regulating the composition of skin secretions (such as antimicrobial peptides and mucus) [93].

Regarding gut microbiota, humidity similarly affects microbial community functions through multiple pathways. High-humidity environments may increase the abundance of moisture-tolerant microorganisms (such as *Bacteroidetes* and *Bacillota*), while low-humidity environments can lead to reduced microbial diversity, particularly among moisture-sensitive taxa (such as *Proteobacteria*) [94]. In addition, in this study, metagenomics and transcriptomics were employed to elucidate the composition and function of amphibian gut microbiota and to explore the impact of environmental factors on microbial communities. The application of these technologies has provided significant insights into the interactions between amphibians and their microbiota. These changes not only impact microbial metabolic functions (such as the production of short-chain fatty acids) but may also affect host health via the “gut–skin axis”. For example, studies on amphibians from the eastern edge of the Tibetan Plateau have shown that low-altitude samples exhibit lower gut microbial diversity and simpler community structures, whereas high-altitude samples display higher diversity and complexity, differences closely associated with humidity gradients [95]. Additionally, research on the Japanese wrinkled frog (*Glandirana rugosa*) has found that hibernation-associated changes in humidity and temperature significantly alter gut microbial community composition and function, possibly related to the host’s reduced metabolic rate and immunosuppressive state [96].

Future research should integrate multi-omics technologies (such as metagenomics, transcriptomics, and metabolomics) to deeply elucidate the mechanisms by which humidity affects microbial communities. Moreover, the long-term impact of humidity interactions with other environmental factors (such as temperature, light, and ultraviolet radiation) on amphibian microbial communities also warrants attention. For example, extreme humidity events caused by climate change may exacerbate microbial community dysbiosis, thereby threatening the survival and population stability of amphibians. In summary, the impact of humidity on amphibian symbiotic microbial communities is multidimensional, involving microbial diversity, functional characteristics, and host–microbe interactions. A deeper understanding of these mechanisms will not only help reveal amphibian adaptation strategies to environmental changes but also provide a scientific basis for amphibian conservation and disease prevention.

### 4.2. Host Factors

Host factors exert selective influences on the composition of symbiotic microbial communities. Research indicates that host factors, including host genotype, gastric pH levels, and antimicrobial peptides, can impose selective pressures on microbial communities, thereby shaping their composition [94]. There is extensive interaction between hosts and their microbial communities; this interplay not only affects the health of the host but may also influence the host’s evolution. Host genetics and their symbiotic microbes are not entirely independent; instead, there is a broad and intricate network of interactions between them [97].

#### 4.2.1. The Impact of Host Specificity on Amphibian Symbiotic Microbiota

The symbiotic microbiota (gut and skin) of hosts exhibit host specificity, meaning there are differences among species; the phylogenetic position of the host affects both the gut and skin microbiota [66]. Different species of amphibians live in various ecological environments and have different needs and choices regarding symbiotic microbiota. For example, some aquatic amphibians may prefer bacteria capable of decomposing organic matter and fixing nitrogen, while terrestrial amphibians may need bacteria capable of decomposing cellulose [98,99]. This host specificity leads to diversity differences in symbiotic microbiota among different species of amphibians.

##### The Impact of Host Specificity on Amphibian Gut Microbiota

Gut microbiota and hosts share a coevolutionary mutualistic relationship [51]. In mammals, the influence of host specificity on gut microbiota is well established. Over time, the lineage and structure of gut microbial communities have coevolved with various mammalian groups, exhibiting diverse variations. Given the significant impact of the gut microbiota on hosts, changes in its composition can drive adaptive evolution in host species, optimizing their integration into the dynamic microbial ecosystem. Consequently, adaptive differences among mammalian species are partly due to variations in gut microbiota composition [100]. However, research on the impact of host specificity on Caudata gut microbiota remains in its infancy, lacking systematic summaries, despite more advanced studies on Anuran gut microbiota.

In Anuran research, significant differences in gut microbiota among different hosts have been observed. Previous studies using 16S rRNA gene sequencing analyzed the gut microbiota of three sympatric frog species (*Odorrana tormota*, *O. graminea*, *Amolops wuyiensis*) [101]. The results indicated significant differences in the relative abundance and predicted functional profiles of gut microbiota among these species, despite relatively similar alpha diversity. Thus, despite comparable alpha diversity and interspecies bacterial similarity, the gut microbiota of these three frog species exhibited substantial differences, potentially linked to bacterial transmission among these anurans. Significant differences in dominant phyla across species further demonstrate that host specificity affects amphibian gut microbiota.

Currently, there is limited research on Gymnophiona, but studies have pointed out significant differences between the gut communities of *Herpele squalostoma* and many frogs and salamanders [102]. The core gut microbial community of adult *H. squalostoma* is dominated by Verrucomicrobia and Tenericutes, which rarely dominate the core gut microbiota of other amphibians. Exploring the symbiotic microbiota of Gymnophiona is not only a key to unlocking the unique biological characteristics of these amphibians but also has significant value in enhancing the ability to protect Gymnophiona

Meanwhile, Nihal Hasan et al. [103] further investigated the underlying reasons for the impact of host specificity on gut microbiota, knowing that host specificity can affect them. Hosts use specific and non-specific factors to cultivate their gut microbiota. Hosts select their gut microbiota by producing several molecular signals that control the surface structures colonized by microbial communities, thereby affecting their composition.

##### The Impact of Host Specificity on Amphibian Skin Microbiota

Different amphibian species may have varying community structures in skin microbiota, as different hosts offer distinct living environments and resources, with only adapted microbiota surviving on their skin [104]. Host specificity can influence microbial function; for instance, some microbiota that survive only on specific amphibian species may help hosts resist pathogens.

Currently, literature on the impact of host specificity on Caudate skin flora is scarce, making it difficult to draw definitive conclusions. However, research on Anurans and other species has been more extensive. Therefore, it is necessary to expand the research scope and focus on exploring the impact of host specificity on the skin microbial communities of Caudates.

There are differences in skin microbiota among Anuran species. Kueneman et al. studied four species cohabiting in California’s Central Valley and found that the bacterial communities on these amphibians’ skin were distinctly different, thus discovering that skin bacterial communities have host species specificity [88]. This conclusion can also be drawn from studies where both Caudate and Anuran are involved. One study found coexisting frogs and salamanders near Braunschweig, Germany, and performed 16SrRNA gene sequencing on their skin microbiota [105]. The results showed that although the skin bacterial richness of frogs and salamanders was similar, their bacterial composition was quite different. The average Jaccard distance between frogs and salamanders exceeded 0.5, while the distances within these groups were only 0.387 and 0.407, respectively. At the OTU level, 31 groups showed significantly different relative abundances between frogs and salamanders, further proving the host specificity of skin microbiota. Similarly, McKenzie et al. reached the same conclusion [89]. They sampled four pond habitats in Colorado, USA, collecting 32 amphibians from three different species: *Lithobates pipiens*, *Pseudacris nigrita*, and *Ambystoma tigrinum*. By barcode pyrosequencing the 16S rRNA gene, they compared the diversity and composition of bacterial communities on the collected individuals’ skin. Dominant bacterial phyla included *Acidobacteria*, *Actinomycetota*, *Bacteroidetes*, *Cyanobacteria*, *Bacillota,* and *Proteobacteria*. Overall, members of 18 bacterial phyla were discovered, comparable to the taxonomic diversity typically found on human skin, with significant differences in bacterial diversity levels among different species. Host specificity affects the composition and richness of amphibian skin microbiota.

In Gymnophiona, host specificity also affects the composition and richness of microbiota. For example, the skin microbial community of *H. squalostoma* shows significant differences compared to many frogs and salamanders. The primary bacterial taxa on the skin of adult *H. squalostoma* do not include Pseudomonadaceae or Moraxellaceae or the genus Pseudomonas, which are typically found to be dominant in the core skin microbiota of frogs and salamanders [102].

Exploring the impact of host specificity on amphibian skin microbiota is crucial for understanding the ecological adaptability and conservation strategies of amphibians. It can help scientists better understand how host genetics shape microbial communities and how microbial composition affects host adaptability, thus offering new perspectives for disease prevention and treatment [106]. Therefore, further exploration in this field will have a profound impact on biodiversity conservation, ecosystem health maintenance, and human well-being enhancement.

#### 4.2.2. The Impact of Host Development on Amphibian Symbiotic Microbiota

Compared to fish, birds, and mammals, amphibians undergo metamorphosis, which involves rapid and drastic changes in the digestive tract and a major dietary shift [107]. During metamorphosis, amphibians transition from larvae to adults, experiencing significant morphological and behavioral changes. For example, frog larvae (tadpoles) live in water and breathe through gills, while adults live on land and primarily breathe through their lungs, with the skin also contributing to respiration. This unique developmental life history impacts the symbiotic microbiota of amphibians in various ways.

##### The Impact of Host Development on Amphibian Gut Microbiota

Vertebrates maintain complex symbiotic relationships with a diverse array of microorganisms within their gastrointestinal tracts. These relationships differ significantly among major vertebrate taxa.

Amphibians undergo metamorphosis during their life cycle, involving rapid and drastic changes in the digestive tract and a major shift in diet. During their lifecycle, amphibians transition through significant morphological and behavioral changes, a process known as metamorphosis. For instance, frog larvae (tadpoles) live in water and breathe through gills, while adults live on land and primarily breathe through lungs, with the skin also contributing to respiration. This unique developmental life history influences the symbiotic microbiota in various ways.

For instance, studies observing the metamorphosis of northern leopard frogs (*Lithobates pipiens*) have revealed significant differences in the gut microbiota between tadpoles and adult frogs. The microbial communities of tadpoles resemble those of fish, whereas those of adult frogs are more similar to those of amniotes. Additionally, compared to tadpoles, adult frogs maintain a lower phylogenetic diversity of their gut microbiota, directly demonstrating the impact of host developmental stage on the gut microbiota [108].

The gut microbiota of amphibians is significantly influenced by the host’s developmental stage, from birth through metamorphosis [109]. During this period, the gut microbiota adapts to new physiological needs and environmental conditions. Dietary shifts, such as from algae to insects, directly alter microbial composition, as different microbes specialize in breaking down various food types. The maturation of the host’s immune system also regulates microbial balance, preventing pathogen overgrowth. Anatomical changes in the gut, like alterations in length and diameter, affect microbial colonization. Increased environmental exposure introduces a broader range of microbes. Collectively, these factors shape the diversity and complexity of the amphibian gut microbiota, influencing host health and adaptation. Thus, understanding the impact of host development on gut microbiota is crucial for elucidating microbe–host interactions and their roles in ecosystems.

##### The Impact of Host Development on Amphibian Skin Microbiota

Developmental stages within the same species can influence the symbiotic microbiota of amphibians. For instance, studies monitoring three amphibian species—Alpine newts (*Ichthyosaura alpestris*), common newts (*Lissotriton vulgaris*), and crested newts (*Triturus cristatus*)—over three months revealed temporal variation in skin-associated microbiota diversity and structural differences between larvae and adults [105]. This transition involves significant skin reshaping and immune function changes [110,111].

Conversely, research on Gymnophiona (*Ichthyophis bannanicus*) found no significant differences in skin microbiota between larvae and adults, likely due to the distinct developmental processes of Gymnophiona compared to other amphibians. Unlike most amphibians that undergo metamorphosis, caecilians exhibit minimal external changes, with juvenile and adult forms being remarkably similar, which may explain the lack of significant differences in skin microbiota across these stages [35].

Observations indicate that amphibians often experience high mortality rates during the transition from larval to adult stages, with certain species suffering severe chytridiomycosis outbreaks shortly after metamorphosis [112]. This period coincides with immune function maturation and bacterial community reorganization on tadpole skin. Thus, immune maturation and skin transformation likely drive changes in skin microbiota. Notably, intense environmental disturbances often correlate with increased pathogen invasion and colonization risks [113].

### 4.3. The Impact of Other Factors on Amphibian Symbiotic Microbiota

In addition to the factors previously mentioned, amphibian symbiotic microbiota are influenced by other variables. For instance, skin microbiota can vary across different body regions, such as ventral and dorsal surfaces. Studies have demonstrated that the ventral α-diversity of the oriental fire-bellied toad (*Bombina orientalis*) exceeds that of the dorsal side [85]. Similarly, in salamanders, which possess toxic steroid alkaloids concentrated in their large parotid glands, significant differences in the composition and diversity of skin bacterial communities have been observed between the dorsal and ventral sides of these glands [114]. Moreover, hibernation impacts the symbiotic microbiota of amphibians. Research on the hibernation of Polypedates megacephalus has shown that gut microbiota composition changes significantly post-hibernation, with frogs subjected to artificial hibernation experiencing an increased susceptibility to bacterial infections [113].

There is also a clear connection between Bd infection and microbial community structure [115]. When amphibians are severely infected with Bd, their skin bacterial communities are severely damaged [116]. Bd infection disrupts the composition of the microbiota and changes the relative abundance of several major bacterial groups [117]. Skin ulcers can also impact the skin microbiota of amphibians. Healthy frogs exhibit higher α-diversity in their skin microbial communities compared to frogs with ulcers. Additionally, the abundance of Proteobacteria and Actinobacteria in the skin microbiota of frogs with ulcers is significantly increased [118]. Light, as one of the environmental factors, significantly influences the skin microbiota of amphibians. Research indicates that light not only directly affects microbial growth and metabolism but also indirectly impacts the structure and function of skin microbial communities by altering the behavior and physiological status of amphibians. For instance, changes in light intensity and photoperiod can alter the activity patterns of amphibians, thereby affecting the frequency and types of contact between their skin and environmental microorganisms [36]. In addition, host genotype [88], gender [86], and other factors can affect symbiotic microbiota.

These factors collectively influence the composition of amphibian symbiotic microbiota. Investigating this relationship is crucial for understanding amphibian health and adaptability. Given the looming threats of severe climate change and pathogens, accelerating research into how microbiota can enhance amphibian resistance is imperative. Moreover, developing microbe-based conservation strategies, such as artificial microbiota transplantation and probiotic therapies, may offer promising approaches for amphibian protection.

## 5. Outlook for Future Research

Based on the foundation of previous work, the study of amphibian symbiotic microbiota should focus on the following aspects: (1) Amphibians, including Caudata, Anura, and Gymnophiona, play a crucial role in ecosystems. However, current research on amphibian symbiotic microbiota is predominantly focused on Caudata and Anura. The Gymnophiona, as an important component of amphibians, has been relatively neglected in terms of symbiotic microbiota research. For instance, studies have shown that the skin microbiota of amphibians, particularly in the Gymnophiona order, can act as the first line of defense against pathogens. Therefore, future research should prioritize the study of symbiotic microbiota in Gymnophiona to enhance our understanding of amphibian symbiotic systems. This will not only reveal the unique microbial community structures and functions of Gymnophiona but also provide a more comprehensive dataset for systematic research on amphibian symbiotic microbiota. (2) The microbial communities in the oral cavity and cloaca play a key role in the development of the amphibian immune system. They establish mutually beneficial relationships with the host, training the host’s immune system to identify and respond to various potential threats. The microbial status of the cloaca may affect the reproductive health of amphibians. Therefore, maintaining the microbial balance of the oral and cloacal cavities of amphibians is crucial for maintaining the health of individual amphibians, promoting their normal physiological functions, and ensuring the health of the entire ecosystem. When studying, protecting, and managing amphibians, the importance of these microbial communities should be emphasized, and research on oral and cloacal microbiota should be strengthened. (3) A database and sample library should be established, and priority should be given to establishing databases and sample libraries of amphibians and their symbiotic microbiota that cover different regions and species, collecting information on amphibians and their symbiotic microbiota. This will provide fundamental data for follow-up research and promote systematic studies on amphibian symbiotic microbiota. (4) The relationship between amphibians and their symbiotic microbiota has gone through a long process of coevolution. Studies have found that specific microbial populations often form stable symbiotic relationships with specific species of amphibians, indicating that there may be signals of coevolution between them. Understanding the evolutionary history of these symbiotic systems helps to reveal the mechanisms of the formation and maintenance of biodiversity. (5) Efforts should be made to strengthen cooperation and exchange with international peers, share research results, learn advanced technologies and concepts, jointly promote the global development of amphibian symbiotic microorganism research, and help protect the biodiversity of amphibians.

## 6. Conclusions

Amphibians, as the most threatened group in vertebrate biodiversity, are increasingly receiving attention for their population diversity. Despite the existing research on the conservation of amphibians, many species are still facing the threat of extinction, especially in the aspects of habitat loss and disease transmission. With the rise of 16S rRNA gene sequencing technology, more and more people have begun to study the amphibian symbiotic microbiota, to understand the functions of symbiotic microbiota and the impact of various factors on symbiotic microbiota, such as environmental factors (temperature, altitude, humidity, etc.), diet, pathogen infection, and host specificity. Future research should integrate multi-omics technologies, such as metagenomics, transcriptomics, and metabolomics, to elucidate the mechanisms by which various factors influence microbial communities. Studying the amphibian symbiotic microbiota helps people understand and protect amphibian biodiversity, maintain the health of the host, and prevent host diseases, while also playing a role in maintaining the stability of the ecosystem.

## Data Availability

The original contributions presented in this study are included in the Supplementary Material. Further inquiries can be directed to the corresponding author.

## Supplementary Materials

The following supporting information can be downloaded at: https://www.mdpi.com/article/10.3390/ani15070934/s1. The references listed in the Supplementary Material are the sources of data for Figure 2 and Figure 3.

## References

[1] Tochimoto T., Shimizu K. (2004). Ecological studies on the Japanese giant salamander, Andrias japonicus, and marking of animals for recognition. Hoshizaki Gurin Zaidan Kenkyu Hokoku.

[2] Liedtke H.C., Wiens J.J., Gomez-Mestre I. (2022). The evolution of reproductive modes and life cycles in amphibians. Nat. Commun..

[3] Fei L., Hu S.Q., Ye C.Y., Huang Y.Z. (2006). Fauna Sinica•Amphibian.

[4] Luedtke J.A., Chanson J., Neam K., Hobin L., Maciel A.O., Catenazzi A., Borzee A., Hamidy A., Aowphol A., Jean A. (2023). Ongoing declines for the world’s amphibians in the face of emerging threats. Nature.

[5] Wake D.B., Vredenburg V.T. (2008). Are we in the midst of the sixth mass extinction? A view from the world of amphibians. Proc. Natl. Acad. Sci. USA.

[6] Xu W., Wu Y.-H., Zhou W.-W., Chen H.-M., Zhang B.-L., Chen J.-M., Xu W., Rao D.-Q., Zhao H., Yan F. (2024). Hidden hotspots of amphibian biodiversity in China. Proc. Natl. Acad. Sci. USA.

[7] Browne R.K., Kaurova S.A., Vasudevan K., McGinnity D., Venu G., Gonzalez M., Uteshev V.K., Marcec-Greaves R. (2022). Reproduction technologies for the sustainable management of Caudata (salamander) and Gymnophiona (caecilian) biodiversity. Reprod. Fertil. Dev..

[8] Alexiev A., Chen M.Y., McKenzie V.J. (2021). Identifying fungal-host associations in an amphibian host system. PLoS ONE.

[9] Roager L., Kempen P.J., Bentzon-Tilia M., Sonnenschein E.C., Gram L. (2024). Impact of host species on assembly, composition, and functional profiles of phycosphere microbiomes. Msystems.

[10] Youngblut N.D., Reischer G.H., Walters W., Schuster N., Walzer C., Stalder G., Ley R.E., Farnleitner A.H. (2019). Host diet and evolutionary history explain different aspects of gut microbiome diversity among vertebrate clades. Nat. Commun..

[11] Wei F.W., Wu Q., Hu Y.B., Huang G.P., Nie Y.G., Yan L. (2019). Conservation metagenomics: A new branch of conservation biology. Sci. China-Life Sci..

[12] Wu Y., Zhuang J., Song Y., Gao X., Chu J., Han S. (2024). Advances in single-cell sequencing technology in microbiome research. Genes Dis..

[13] Bletz M.C., Perl R.G.B., Bobowski B.T.C., Japke L.M., Tebbe C.C., Dohrmann A.B., Bhuju S., Geffers R., Jarek M., Vences M. (2017). Amphibian skin microbiota exhibits temporal variation in community structure but stability of predicted *Bd*-inhibitory function. ISME J..

[14] Harris R.N., Brucker R.M., Walke J.B., Becker M.H., Schwantes C.R., Flaherty D.C., Lam B.A., Woodhams D.C., Briggs C.J., Vredenburg V.T. (2009). Skin microbes on frogs prevent morbidity and mortality caused by a lethal skin fungus. ISME J..

[15] Zhu W., Chang L., Shi S., Lu N., Du S., Li J., Jiang J., Wang B. (2024). Gut microbiota reflect adaptation of cave-dwelling tadpoles to resource scarcity. ISME J..

[16] Brunetti A.E., Lyra M.L., Melo W.G.P., Andrade L.E., Palacios-Rodríguez P., Prado B.M., Haddad C.F.B., Pupo M.T., Lopes N.P. (2019). Symbiotic skin bacteria as a source for sex-specific scents in frogs. Proc. Natl. Acad. Sci. USA.

[17] Zhu W., Zhao C., Feng J., Chang J., Zhu W., Chang L., Liu J., Xie F., Li C., Jiang J. (2022). Effects of Habitat River Microbiome on the Symbiotic Microbiota and Multi-Organ Gene Expression of Captive-Bred Chinese Giant Salamander. Front. Microbiol..

[18] Zhu B., Shao C., Xu W.J., Dai J.H., Fu G.H., Hu Y. (2024). Effects of Thyroid Powder on Tadpole (*Lithobates catesbeiana*) Metamorphosis and Growth: The Role of Lipid Metabolism and Gut Microbiota. Animals.

[19] Tong Q., Hu Z.F., Du X.P., Bie J., Wang H.B. (2020). Effects of Seasonal Hibernation on the Similarities Between the Skin Microbiota and Gut Microbiota of an Amphibian (Rana dybowskii). Microb. Ecol..

[20] Dallas J.W., Meshaka W.E., Zeglin L., Warne R.W. (2021). Taxonomy, not locality, influences the cloacal microbiota of two nearctic colubrids: A preliminary analysis. Mol. Biol. Rep..

[21] McFall-Ngai M., Hadfield M.G., Bosch T.C.G., Carey H.V., Domazet-Loso T., Douglas A.E., Dubilier N., Eberl G., Fukami T., Gilbert S.F. (2013). Animals in a bacterial world, a new imperative for the life sciences. Proc. Natl. Acad. Sci. USA.

[22] Vos W.M.D., Tilg H., Hul M.V., Cani P.D. (2022). Gut microbiome and health: Mechanistic insights. Gut.

[23] Lize A., McKay R., Lewis Z. (2013). Gut microbiota and kin recognition. Trends Ecol. Evol..

[24] Zhu W., Chang L., Zhang M., Chen Q., Sui L., Shen C., Jiang J. (2024). Microbial diversity in mountain-dwelling amphibians: The combined effects of host and climatic factors. iScience.

[25] Lee Y.Y., Hassan S.A., Ismail I.H., Chong S.Y., Ali R.A.R., Nordin S.A., Lee W.S., Majid N.A. (2017). Gut microbiota in early life and its influence on health and disease: A position paper by the Malaysian Working Group on Gastrointestinal Health. J. Paediatr. Child Health.

[26] Anand S., Mande S.S. (2022). Host-microbiome interactions: Gut-Liver axis and its connection with other organs. Npj Biofilms Microbiomes.

[27] Austin R.M. Cutaneous microbial flora and antibiosis in *Plethodon ventralis* -: Inferences for parental care in the Plethodontidae. Proceedings of the 4th Conference on the Biology of Plethodontid Salamanders.

[28] Jimenez R.R., Sommer S. (2017). The amphibian microbiome: Natural range of variation, pathogenic dysbiosis, and role in conservation. Biodivers. Conserv..

[29] Varki A. (1993). Biological Roles of Oligosaccharides—All of the Theories Are Correct. Glycobiology.

[30] Woodhams D.C., Bletz M., Kueneman J., McKenzie V. (2016). Managing Amphibian Disease with Skin Microbiota. Trends Microbiol..

[31] Ross A.A., Hoffmann A.R., Neufeld J.D. (2019). The skin microbiome of vertebrates. Microbiome.

[32] Woodhams D.C., Geiger C.C., Reinert L.K., Rollins-Smith L.A., Lam B., Harris R.N., Briggs C.J., Vredenburg V.T., Voyles J. (2012). Treatment of amphibians infected with chytrid fungus: Learning from failed trials with itraconazole, antimicrobial peptides, bacteria, and heat therapy. Dis. Aquat. Org..

[33] Bartos O., Chmel M., Swierczkova I. (2024). The overlooked evolutionary dynamics of 16S rRNA revises its role as the “gold standard” for bacterial species identification. Sci. Rep..

[34] Li M.-N., Han Q., Wang N., Wang T., You X.-M., Zhang S., Zhang C.-C., Shi Y.-Q., Qiao P.-Z., Man C.-L. (2024). 16S rRNA gene sequencing for bacterial identification and infectious disease diagnosis. Biochem. Biophys. Res. Commun..

[35] Wu Z.L. (2022). Analysis of symbiotic microbe community on the Ichthyophis bannanicus. Master’s Thesis.

[36] Hernandez-Gomez O., Hua J. (2023). From the organismal to biosphere levels: Environmental impacts on the amphibian microbiota. Fems Microbiol. Rev..

[37] Fontaine S.S., Novarro A.J., Kohl K.D. (2018). Environmental temperature alters the digestive performance and gut microbiota of a terrestrial amphibian. J. Exp. Biol..

[38] Küng D., Bigler L., Davis L.R., Gratwicke B., Griffith E., Woodhams D.C. (2014). Stability of Microbiota Facilitated by Host Immune Regulation: Informing Probiotic Strategies to Manage Amphibian Disease. PLoS ONE.

[39] Rebollar E.A., Hughey M.C., Medina D., Harris R.N., Ibáñez R., Belden L.K. (2016). Skin bacterial diversity of Panamanian frogs is associated with host susceptibility and presence of Batrachochytrium dendrobatidis. ISME J..

[40] Walke J.B., Becker M.H., Loftus S.C., House L.L., Cormier G., Jensen R.V., Belden L.K. (2014). Amphibian skin may select for rare environmental microbes. ISME J..

[41] Loudon A.H., Woodhams D.C., Parfrey L.W., Archer H., Knight R., McKenzie V., Harris R.N. (2014). Microbial community dynamics and effect of environmental microbial reservoirs on red-backed salamanders (*Plethodon cinereus*). ISME J..

[42] Varela B.J., Lesbarrères D., Ibáñez R., Green D.M. (2018). Environmental and Host Effects on Skin Bacterial Community Composition in Panamanian Frogs. Front. Microbiol..

[43] Chang C.W., Huang B.H., Lin S.M., Huang C.L., Liao P.C. (2016). Changes of diet and dominant intestinal microbes in farmland frogs. Bmc Microbiol..

[44] Huang S.W., Zhang H.Y. (2013). The Impact of Environmental Heterogeneity and Life Stage on the Hindgut Microbiota of *Holotrichia parallela* Larvae (*Coleoptera: Scarabaeidae*). PLoS ONE.

[45] Ren C.L. (2022). Effects of Temperature on Intestinal Microbiota, Lipid Metabolism, and Skeletal Development in Chinese Brown Frogs. Master’s Thesis.

[46] Emerson K.J., Fontaine S.S., Kohl K.D., Woodley S.K. (2023). Temperature and the microbial environment alter brain morphology in a larval amphibian. J. Exp. Biol..

[47] Wiggins P.J., Smith J.M., Harris R.N., Minbiole K.P.C. (2011). Gut of Red-backed Salamanders (*Plethodon cinereus*) May Serve as a Reservoir for an Antifungal Cutaneous Bacterium. J. Herpetol..

[48] Antwis R.E., Haworth R.L., Engelmoer D.J.P., Ogilvy V., Fidgett A.L., Preziosi R.F. (2014). Ex situ Diet Influences the Bacterial Community Associated with the Skin of Red-Eyed Tree Frogs (*Agalychnis callidryas*). PLoS ONE.

[49] Lauer A., Simon M.A., Banning J.L., André E., Duncan K., Harris R.N. (2007). Common cutaneous bacteria from the eastern red-backed salamander can inhibit pathogenic fungi. Copeia.

[50] Colombo B.M., Scalvenzi T., Benlamara S., Pollet N. (2015). Microbiota and mucosal immunity in amphibians. Front. Immunol..

[51] Sanders J.G., Powell S., Kronauer D.J.C., Vasconcelos H.L., Frederickson M.E., Pierce N.E. (2014). Stability and phylogenetic correlation in gut microbiota: Lessons from ants and apes. Mol. Ecol..

[52] Lima L.F.O., Weissman M., Reed M., Papudeshi B., Alker A.T., Morris M.M., Edwards R.A., de Putron S.J., Vaidya N.K., Dinsdale E.A. (2020). Modeling of the Coral Microbiome: The Influence of Temperature and Microbial Network. Mbio.

[53] Zhao J., Xie X., Jiang Y., Li J., Fu Q., Qiu Y., Fu X., Yao Z., Dai Z., Qiu Y. (2024). Effects of simulated warming on soil microbial community diversity and composition across diverse ecosystems. Sci. Total Environ..

[54] Zhao P., Huang Y., Liu B., Chen J., Lei Z., Zhang Y., Cheng B., Zhou T., Peng S. (2024). Effects of daytime and nighttime warming on soil microbial diversity. Geoderma.

[55] Li J., Rui J., Li Y., Tang N., Zhan S., Jiang J., Li X. (2020). Ambient temperature alters body size and gut microbiota of Xenopus tropicalis. Sci. China-Life Sci..

[56] Huey R.B. (1991). Physiological Consequences of Habitat Selection. Am. Nat..

[57] Rowley J.J.L., Alford R.A. (2013). Hot bodies protect amphibians against chytrid infection in nature. Sci. Rep..

[58] Meyer E.A., Cramp R.L., Bernal M.H., Franklin C.E. (2012). Changes in cutaneous microbial abundance with sloughing: Possible implications for infection and disease in amphibians. Dis. Aquat. Org..

[59] Ohmer M.E.B., Cramp R.L., White C.R., Franklin C.E. (2015). Skin sloughing rate increases with chytrid fungus infection load in a susceptible amphibian. Funct. Ecol..

[60] Woodhams D.C., Brandt H., Baumgartner S., Kielgast J., Küpfer E., Tobler U., Davis L.R., Schmidt B.R., Bel C., Hodel S. (2014). Interacting Symbionts and Immunity in the Amphibian Skin Mucosome Predict Disease Risk and Probiotic Effectiveness. PLoS ONE.

[61] Daskin J.H., Bell S.C., Schwarzkopf L., Alford R.A. (2014). Cool Temperatures Reduce Antifungal Activity of Symbiotic Bacteria of Threatened Amphibians—Implications for Disease Management and Patterns of Decline. PLoS ONE.

[62] Muletz-Wolz C.R., Fleischer R.C., Lips K.R. (2019). Fungal disease and temperature alter skin microbiome structure in an experimental salamander system. Mol. Ecol..

[63] Troitsky T.S., Laine V.N., Lilley T.M. (2023). When the host’s away, the pathogen will play: The protective role of the skin microbiome during hibernation. Anim. Microbiome.

[64] Bresciano J.C., Salvador C.A., Paz-y-Miño C., Parody-Merino A.M., Bosch J., Woodhams D.C. (2015). Variation in the Presence of Anti-*Batrachochytrium dendrobatidis* Bacteria of Amphibians Across Life Stages and Elevations in Ecuador. Ecohealth.

[65] Wang J.J., Soininen J., Zhang Y., Wang B.X., Yang X.D., Shen J. (2011). Contrasting patterns in elevational diversity between microorganisms and macroorganisms. J. Biogeogr..

[66] Xu L.L. (2019). The Impact of Altitude on Amphibian Symbiotic Microbiota (Skin and Gut Microbiomes). Master’s Thesis.

[67] Zhao J.S., Yao Y.F., Li D.Y., Xu H.M., Wu J.Y., Wen A.X., Xie M., Ni Q.Y., Zhang M.W., Peng G.N. (2018). Characterization of the Gut Microbiota in Six Geographical Populations of Chinese Rhesus Macaques (*Macaca mulatta*), Implying an Adaptation to High-Altitude Environment. Microb. Ecol..

[68] Zhang Z.G., Xu D.M., Wang L., Hao J.J., Wang J.F., Zhou X., Wang W.W., Qiu Q., Huang X.D., Zhou J.W. (2016). Convergent Evolution of Rumen Microbiomes in High-Altitude Mammals. Curr. Biol..

[69] Satanower S. (2022). Microbiome Research: An Overview. Aldrichimica Acta.

[70] Guo J., Zhao R., Li K., Tan Y., Wang L., Ling H., Zhang H., Dharmarajan G., Bi Y., Yang R. (2025). Altitude adaptation: The unseen work of gut microbiota. hLife.

[71] Muletz Wolz C.R., Yarwood S.A., Campbell Grant E.H., Fleischer R.C., Lips K.R., Hoye B. (2017). Effects of host species and environment on the skin microbiome of Plethodontid salamanders. J. Anim. Ecol..

[72] Zeng B., Zhao J.C., Guo W., Zhang S.Y., Hua Y.T., Tang J.S., Kong F.L., Yang X.W., Fu L.Z., Liao K. (2017). High-Altitude Living Shapes the Skin Microbiome in Humans and Pigs. Front. Microbiol..

[73] Zhou J., Liu Z., Wang S., Li J., Zhang L., Liao Z. (2025). A novel framework unveiling the importance of heterogeneous selection and drift on the community structure of symbiotic microbial indicator taxa across altitudinal gradients in amphibians. Microbiol. Spectr..

[74] Secor S.M., Carey H.V. (2016). Integrative Physiology of Fasting. Compr. Physiol..

[75] Carey H.V., Assadi-Porter F.M., Stover P.J., Balling R. (2017). The Hibernator Microbiome: Host-Bacterial Interactions in an Extreme Nutritional Symbiosis. Annual Review of Nutrition.

[76] Dai Y.F. (2024). Research Progress on the Functional Regulation of Intestinal Microbial in Chinese Giant Salamander. Adv. Microbiol..

[77] Lemieux-Labonté V., Vigliotti C., Tadic Z., Wehrle B., Lopez P., Bapteste E., Lapointe F.J., German D.P., Herrel A. (2022). Proximate Drivers of Population-Level Lizard Gut Microbial Diversity: Impacts of Diet, Insularity, and Local Environment. Microorganisms.

[78] Kohl K.D., Yahn J. (2016). Effects of environmental temperature on the gut microbial communities of tadpoles. Environ. Microbiol..

[79] Hou K., Wu Z.-X., Chen X.-Y., Wang J.-Q., Zhang D., Xiao C., Zhu D., Koya J.B., Wei L., Li J. (2022). Microbiota in health and diseases. Signal Transduct. Target. Ther..

[80] Wang Y., Xie Y., Wu S., Zhang W., Cheng X., Li Z., Han F., Shi J., Shi Y., He Z. (2024). Effects of Dietary Changes on the Gut Microbiota of Cynops orientalis. Asian Herpetol. Res..

[81] Tong Q., Liu X.-N., Hu Z.-F., Ding J.-F., Bie J., Wang H.-B., Zhang J.-T. (2019). Effects of Captivity and Season on the Gut Microbiota of the Brown Frog (*Rana dybowskii*). Front. Microbiol..

[82] Knutie S.A., Shea L.A., Kupselaitis M., Wilkinson C.L., Kohl K.D., Rohr J.R. (2017). Early-Life Diet Affects Host Microbiota and Later-Life Defenses Against Parasites in Frogs. Integr. Comp. Biol..

[83] Demircan T., Ovezmyradov G., Yildirim B., Keskin I., Ilhan A.E., Fescioglu E.C., Ozturk G., Yildirim S. (2018). Experimentally induced metamorphosis in highly regenerative axolotl (*ambystoma mexicanum*) under constant diet restructures microbiota. Sci. Rep..

[84] Yang B., Cui Z., Ning M., Chen Y., Wu Z., Huang H. (2022). Variation in the intestinal microbiota at different developmental stages of *Hynobius maoershanensis*. Ecol. Evol..

[85] Sabino-Pinto J., Bletz M.C., Islam M.M., Shimizu N., Bhuju S., Geffers R., Jarek M., Kurabayashi A., Vences M. (2016). Composition of the Cutaneous Bacterial Community in Japanese Amphibians: Effects of Captivity, Host Species, and Body Region. Microb. Ecol..

[86] Hernández-Gómez O., Briggler J.T., Williams R.N. (2018). Captivity-Induced Changes in the Skin Microbial Communities of Hellbenders (Cryptobranchus alleganiensis). Microb. Ecol..

[87] Wilson B.A., Passos L.F., Garcia G., Young R.J. (2018). Comparing the bacterial communities of wild and captive golden mantella frogs: Implications for amphibian conservation. PLoS ONE.

[88] Kueneman J.G., Parfrey L.W., Woodhams D.C., Archer H.M., Knight R., McKenzie V.J. (2014). The amphibian skin-associated microbiome across species, space and life history stages. Mol. Ecol..

[89] McKenzie V.J., Bowers R.M., Fierer N., Knight R., Lauber C.L. (2012). Co-habiting amphibian species harbor unique skin bacterial communities in wild populations. ISME J..

[90] Vences M., Dohrmann A.B., Künzel S., Granzow S., Baines J.F., Tebbe C.C. (2015). Composition and variation of the skin microbiota in sympatric species of European newts (Salamandridae). Amphib. -Reptil..

[91] Bates K.A., Friesen J., Loyau A., Butler H., Vredenburg V.T., Laufer J., Chatzinotas A., Schmeller D.S. (2022). Environmental and Anthropogenic Factors Shape the Skin Bacterial Communities of a Semi-Arid Amphibian Species. Microb. Ecol..

[92] Lucia Z., Giulio G., Matteo G., Stefano C., Irene L.P., Paolo P., Giorgio B., Hauffe H.C. (2024). More Than Meets the Eye: Unraveling the Interactions Between Skin Microbiota and Habitat in an Opportunistic Amphibian. Microb. Ecol..

[93] Zhang J.P. (2013). *Rana dybowskii* Surface Bacteria Identification and the Habitat Microbe Analysis. Master’s Thesis.

[94] Bletz M.C., Goedbloed D.J., Sanchez E., Reinhardt T., Tebbe C.C., Bhuju S., Geffers R., Jarek M., Vences M., Steinfartz S. (2016). Amphibian gut microbiota shifts differentially in community structure but converges on habitat-specific predicted functions. Nat. Commun..

[95] Xu L.L., Chen H., Zhang M., Zhu W., Chang Q., Lu G., Chen Y., Jiang J., Zhu L. (2020). Changes in the community structure of the symbiotic microbes of wild amphibians from the eastern edge of the Tibetan Plateau. Microbiologyopen.

[96] Park J.-K., Do Y. (2024). The difference and variation of gut bacterial community and host physiology can support adaptation during and after overwintering in frog population. Integr. Zool..

[97] Zhu Z., Liu Y., Hu H., Wang G.-H. (2023). *Nasonia*-microbiome associations: A model for evolutionary hologenomics research. Trends Parasitol..

[98] Korpita T.M.M., Muths E.L.L., Watry M.K., McKenzie V.J.J. (2023). Captivity, Reintroductions, and the Rewilding of Amphibian-associated Bacterial Communities. Microb. Ecol..

[99] Wang C.L., Chen H., Xiao H., Zhang H.M., Li L.Z., Guo C., Chen J., Wei Q. (2020). Amphibian diversity and habitat selection in karst desertification areas of northwest Guizhou. Biodivers. Sci..

[100] Cani P.D., Delzenne N.M., Amar J., Burcelin R. (2008). Role of gut microflora in the development of obesity and insulin resistance following high-fat diet feeding. Pathol. Biol..

[101] Chen Z., Chen J.-Q., Liu Y., Zhang J., Chen X.-H., Qu Y.-F. (2022). Comparative study on gut microbiota in three Anura frogs from a mountain stream. Ecol. Evol..

[102] Kouete M.T., Bletz M.C., LaBumbard B.C., Woodhams D.C., Blackburn D.C. (2023). Parental care contributes to vertical transmission of microbes in a skin-feeding and direct-developing caecilian. Anim. Microbiome.

[103] Hasan N., Yang H.Y. (2019). Factors affecting the composition of the gut microbiota, and its modulation. Peerj.

[104] Bird A.K., Prado-Irwin S.R., Vredenburg V.T., Zink A.G. (2018). Skin Microbiomes of California Terrestrial Salamanders Are Influenced by Habitat More Than Host Phylogeny. Front. Microbiol..

[105] Bletz M.C., Perl R.G.B., Vences M. (2017). Skin microbiota differs drastically between co-occurring frogs and newts. R. Soc. Open Sci..

[106] Chen Y.E., Fischbach M.A., Belkaid Y. (2018). Skin microbiota–host interactions. Nature.

[107] Weng F.C.-H., Yang Y.-J., Wang D. (2016). Functional analysis for gut microbes of the brown tree frog (*Polypedates megacephalus*) in artificial hibernation. BMC Genom..

[108] Kohl K.D., Cary T.L., Karasov W.H., Dearing M.D. (2013). Restructuring of the amphibian gut microbiota through metamorphosis. Environ. Microbiol. Rep..

[109] Weng F.C.-H., Shaw G.T.-W., Weng C.-Y., Yang Y.-J., Wang D. (2017). Inferring Microbial Interactions in the Gut of the Hong Kong Whipping Frog (*Polypedates megacephalus*) and a Validation Using Probiotics. Front. Microbiol..

[110] Faszewski E.E., Kaltenbach J.C. (1995). Histology and lectin-binding patterns in the skin of the terrestrial horned frog ceratophrys ornata. Cell Tissue Res..

[111] Robinson D.H., Heintzelman M.B. (1987). Morphology of ventral epidermis of rana-catesbeiana during metamorphosis. Anat. Rec..

[112] Bosch J., Martínez-Solano I., García-París M. (2001). Evidence of a chytrid fungus infection involved in the decline of the common midwife toad (*Alytes obstetricans*) in protected areas of central Spain. Biol. Conserv..

[113] Shade A., Peter H., Allison S.D., Baho D.L., Berga M., Buergmann H., Huber D.H., Langenheder S., Lennon J.T., Martiny J.B.H. (2012). Fundamentals of microbial community resistance and resilience. Front. Microbiol..

[114] Van Veelen H.P.J., Salles J.F., Matson K.D., van der Velde M., Tieleman B.I. (2020). Microbial environment shapes immune function and cloacal microbiota dynamics in zebra finches Taeniopygia guttata. Anim. Microbiome.

[115] Kruger A. (2020). Frog Skin Microbiota Vary with Host Species and Environment but Not Chytrid Infection. Front. Microbiol..

[116] Bataille A., Lee-Cruz L., Tripathi B., Kim H., Waldman B. (2016). Microbiome Variation Across Amphibian Skin Regions: Implications for Chytridiomycosis Mitigation Efforts. Microb. Ecol..

[117] Jani A.J., Bushell J., Arisdakessian C.G., Belcaid M., Boiano D.M., Brown C., Knapp R.A. (2021). The amphibian microbiome exhibits poor resilience following pathogen-induced disturbance. ISME J..

[118] Zhu D.-q., Dong W.-j., Long X.-z., Yang X.-m., Han X.-y., Kou Y.-h., Tong Q. (2024). Skin ulcers and microbiota in Rana dybowskii: Uncovering the role of the gut-skin axis in amphibian health. Aquaculture.

